# Author Correction: Lifespan-extending interventions induce consistent patterns of fatty acid oxidation in mouse livers

**DOI:** 10.1038/s42003-023-05549-9

**Published:** 2023-11-27

**Authors:** Kengo Watanabe, Tomasz Wilmanski, Priyanka Baloni, Max Robinson, Gonzalo G. Garcia, Michael R. Hoopmann, Mukul K. Midha, David H. Baxter, Michal Maes, Seamus R. Morrone, Kelly M. Crebs, Charu Kapil, Ulrike Kusebauch, Jack Wiedrick, Jodi Lapidus, Lance Pflieger, Christopher Lausted, Jared C. Roach, Gwênlyn Glusman, Steven R. Cummings, Nicholas J. Schork, Nathan D. Price, Leroy Hood, Richard A. Miller, Robert L. Moritz, Noa Rappaport

**Affiliations:** 1 Institute for Systems Biology, Seattle, WA USA; 2 School of Health Sciences, Purdue University, West Lafayette, IN USA; 3 Department of Pathology, University of Michigan School of Medicine, Ann Arbor, MI USA; 4 Oregon Health and Science University, Portland, OR USA; 5 Phenome Health, Seattle, WA USA; 6 San Francisco Coordinating Center, California Pacific Medical Center Research Institute, San Francisco, CA USA; 7 Department of Epidemiology and Biostatistics, University of California, San Francisco, CA USA; 8 Department of Quantitative Medicine, The Translational Genomics Research Institute (TGen), Phoenix, AZ USA; 9 Department of Population Sciences and Molecular and Cell Biology, The City of Hope National Medical Center, Duarte, CA USA; 10 Thorne HealthTech, New York, NY USA; 11 Department of Bioengineering, University of Washington, Seattle, WA USA; 12 Paul G. Allen School of Computer Science & Engineering, University of Washington, Seattle, WA USA; 13 Department of Immunology, University of Washington, Seattle, WA USA; 14 University of Michigan Geriatrics Center, Ann Arbor, MI USA

**Keywords:** Systems biology, Computational biology and bioinformatics, Proteomics

Correction to: *Communications Biology* 10.1038/s42003-023-05128-y, published online 22 July 2023

In this article, one of the affiliation details for Richard A. Miller was incorrectly given as ‘School of Health Sciences, Purdue University, West Lafayette, IN, USA’ but should have been ‘Department of Pathology, University of Michigan School of Medicine, Ann Arbor, MI, USA’. The original article has been corrected.

