# Supplementary material for: Lifespan-extending interventions induce consistent patterns of fatty acid oxidation in mouse livers
Source: Commun Biol. 2023 Jul 22;6:768. doi: 10.1038/s42003-023-05128-y (PMC10363145; doi:10.1038/s42003-023-05128-y)
Supplement: Supplementary file 2 — Supplementary Information [file 42003_2023_5128_MOESM2_ESM.pdf]

Supplementary Information

Lifespan-extending interventions induce consistent patterns of fatty acid oxidation in mouse livers

Kengo Watanabe, Tomasz Wilmanski, Priyanka Baloni, Max Robinson, Gonzalo G. Garcia, Michael R. Hoopmann, Mukul K. Midha, David H. Baxter, Michal Maes, Seamus R. Morrone, Kelly M. Crebs, Charu Kapil, Ulrike Kusebauch, Jack Wiedrick, Jodi Lapidus, Lance Pflieger, Christopher Lausted, Jared C. Roach, Gwênlyn Glusman, Steven R. Cummings, Nicholas J. Schork, Nathan D. Price, Leroy Hood, Richard A. Miller, Robert L. Moritz, and Noa Rappaport

Correspondence: Leroy Hood (lee.hood@isbscience.org), Noa Rappaport (noa.rappaport@isbscience.org)

This PDF file includes:

Supplementary Figures 1 to 4

Other Supplementary Information files in this study include the followings:

Supplementary Data 1 to 9

Supplementary Figures

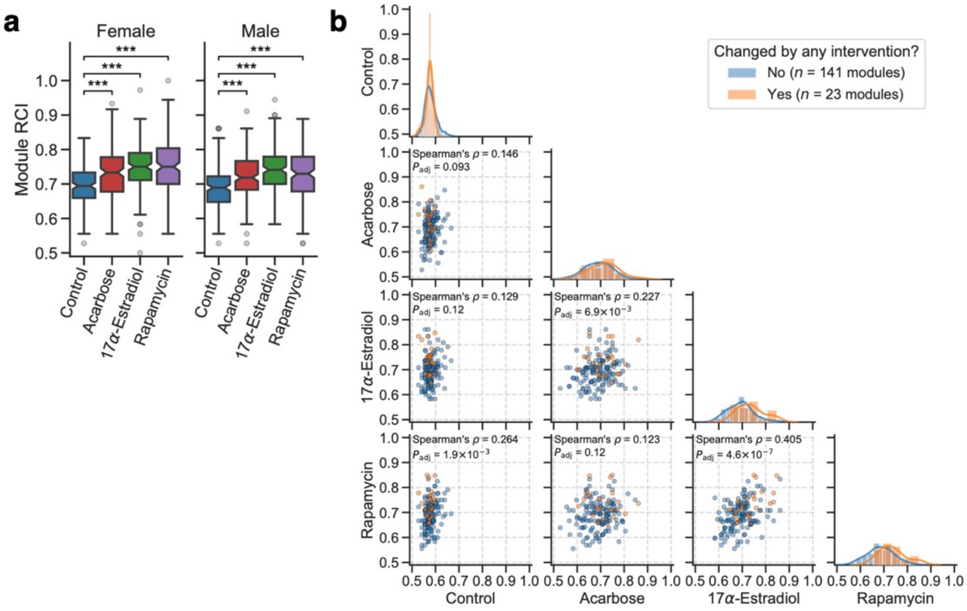

Supplementary Figure 1. Supplementary analysis about the module RCI distributions.

a Overall distribution of module rank conservation index (RCI) with sex stratification. Differential Rank Conservation (DIRAC) analysis of the LC-M001 proteomics data using Gene Ontology Biological Process (GOBP)-defined modules was re-performed, except for using the unadjusted data (i.e., the data before the potential effects of sex were regressed out) and sex-stratified sample groups. Data: median (center line), 95% confidence interval (CI) around median (notch), [Q_1, Q_3] (box limits), [$x_{\min}$, $x_{\max}$] (whiskers), where Q_1 and Q_3 are the 1st and 3rd quartile values, and $x_{\min}$ and $x_{\max}$ are the minimum and maximum values in [$Q_1 - 1.5 \times$ IQR, $Q_3 + 1.5 \times$ IQR] (IQR: the interquartile range, $Q_3 - Q_1$), respectively; $n = 164$ modules. ***$P < 0.001$ by two-sided Mann–Whitney U-tests after the Benjamini–Hochberg adjustment across six (three comparisons × two sexes) comparisons. **b** Pairwise correlations of module RCI among the sample groups. Shown are the result from DIRAC analysis of the LC-M001 proteomics data using GOBP-defined modules, corresponding to Fig. 2. P_{adj}: P-value in Spearman's correlation test after the Benjamini–Hochberg adjustment across six pairs. $n = 164$ modules. The line in the histogram panel indicates the kernel density estimate.

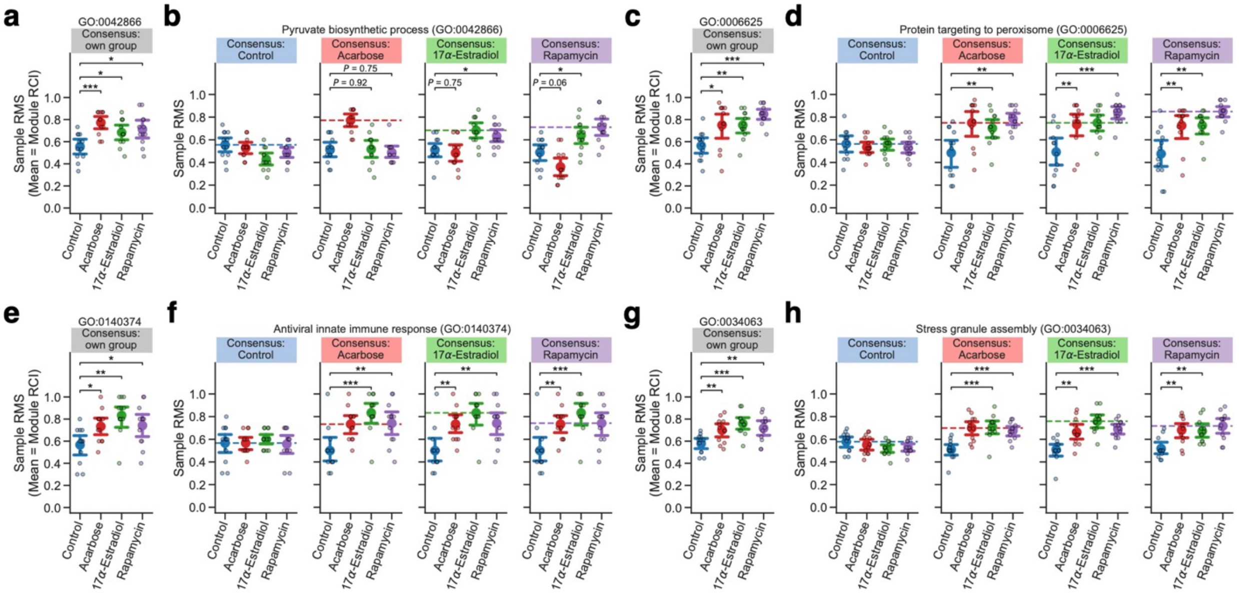

Supplementary Figure 2. Examples of the tightened proteomic modules by lifespan-extending interventions.

Shown are the results from Differential Rank Conservation (DIRAC) analysis of the LC-M001 proteomics data using Gene Ontology Biological Process (GOBP)-defined modules, corresponding to Fig. 2. **a**–**h** Sample rank matching score (RMS) distributions for examples of the tightly but differently tightened modules between intervention groups (**a**, **b**; GO:0042866, pyruvate biosynthetic process) or the similarly tightened modules (**c**–**h**; GO:0006625, protein targeting to peroxisome; GO:0140374, antiviral innate immune response; GO:0034063, stress granule assembly). Dashed line in **b**, **d**, **f**, and **h** indicates the mean of RMSs for the rank consensus group (i.e., rank conservation index, RCI). Data: the mean (dot) with 95% confidence interval (CI) (bar); $n = 12$ mice. *$P < 0.05$, **$P < 0.01$, ***$P < 0.001$ by two-sided Welch's t-tests after the Benjamini–Hochberg adjustment across three (**a**, **c**, **e**, **g**) or six (two comparisons × three rank consensus; **b**, **d**, **f**, **h**) comparisons.

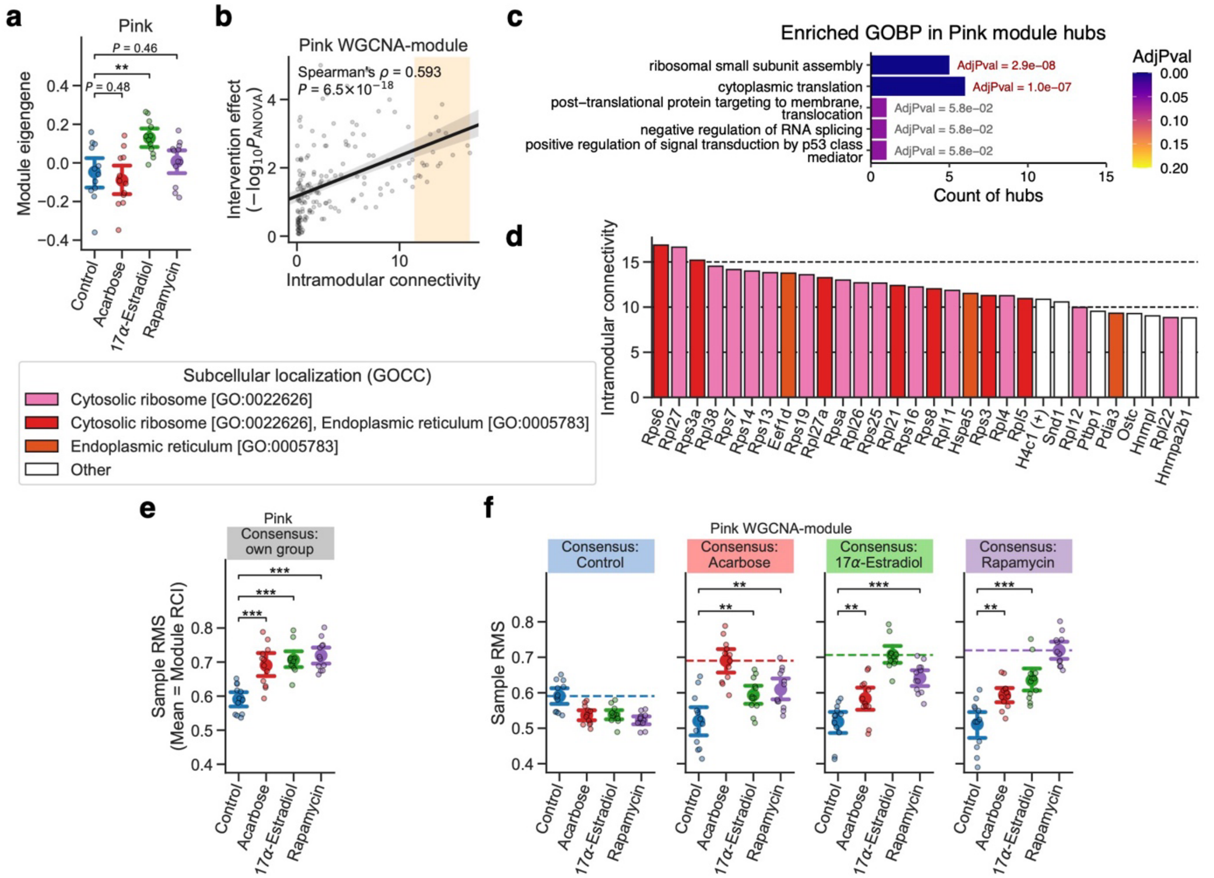

Supplementary Figure 3. WGCNA and DIRAC results of the Pink module.

Shown are the results from Weighted Gene Co-expression Network Analysis (WGCNA) of the LC-M001 proteomics data (**a**–**d**) and the results from Differential Rank Conservation (DIRAC) analysis of the LC-M001 proteomics data using WGCNA-identified modules (**e**, **f**), corresponding to Fig. 3. **a** Distributions of sample's module eigengene values for the Pink module. Data: the mean (dot) with 95% confidence interval (CI) (bar); $n = 12$ mice. $^{**}P < 0.01$ by two-sided Welch's t-tests after the Benjamini–Hochberg adjustment across three comparisons. **b** Relationship between the intervention effect on each protein and their respective intramodular connectivity in the Pink module. The P-value of y-axis corresponds to the main effect of intervention on each protein level by Analysis of Variance (ANOVA). The line is the ordinary least squares (OLS) linear regression line with 95% CI, and the orange-colored background reflects the range of top 10% hub proteins (18 proteins). $n = 174$ proteins. **c** Enriched Gene Ontology Biological Process (GOBP) terms in the top 10% hub proteins of the Pink module. Significance was assessed using overrepresentation tests after the Benjamini–Hochberg adjustment across seven terms. Only the GOBP terms that exhibited nominal $P < 0.05$ are presented. AdjPval: adjusted P-value from the overrepresentation test. **d** Top 30 hub proteins of the Pink module. GOCC: GO Cellular Component. **e**, **f** Sample rank matching score (RMS) distributions for the Pink module. Dashed line in **f** indicates the mean of RMSs for the rank consensus group (i.e., rank conservation index, RCI). Data: the mean (dot) with 95% CI (bar); $n = 12$ mice. $^{**}P < 0.01$, $^{***}P < 0.001$ by two-sided Welch's t-tests after the Benjamini–Hochberg adjustment across three (**e**) or six (two comparisons × three rank consensus; **f**) comparisons.

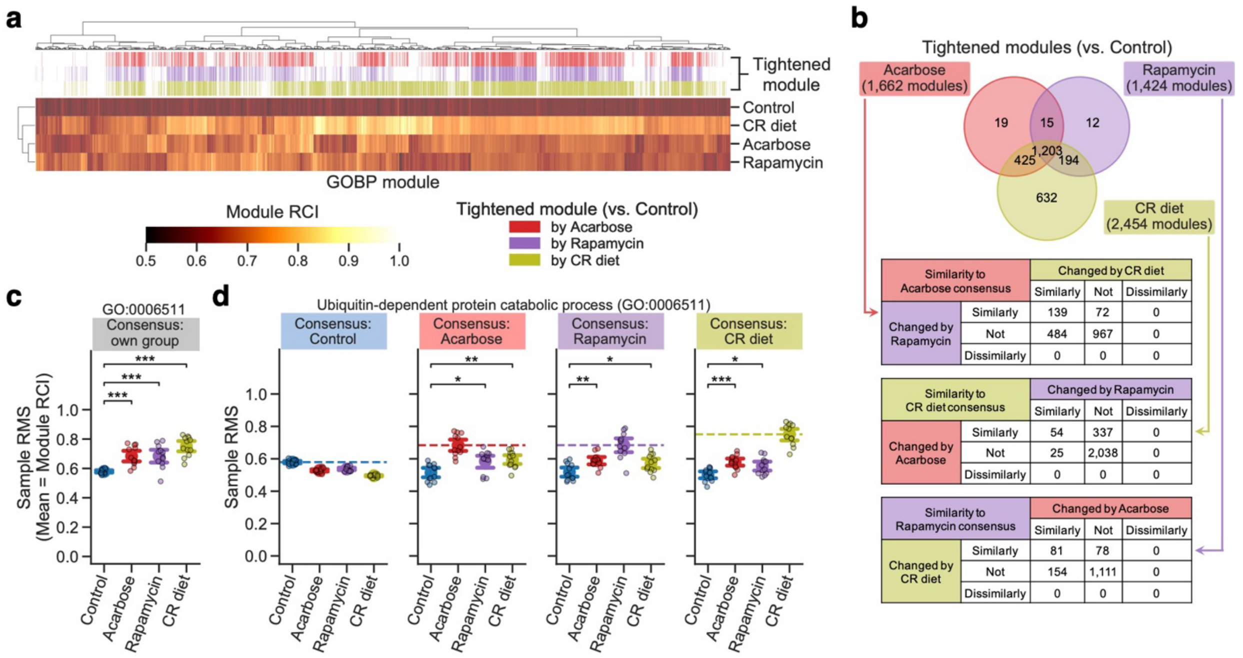

Supplementary Figure 4. DIRAC results of the M001-related transcriptomics data.

a–**d** Differential Rank Conservation (DIRAC) analysis of the M001-related transcriptomics data using Gene Ontology Biological Process (GOBP)-defined modules, corresponding to Fig. 4a. **a** Overall distribution of module rank conservation index (RCI). The top color columns highlight the modules that exhibited (1) the significant intervention effect on module RCI (Analysis of Variance (ANOVA) after the Benjamini–Hochberg adjustment across 3,912 modules) and (2) significantly higher RCI in intervention group than control group (i.e., tightened module; the post hoc two-sided Welch's *t*-tests after the Benjamini–Hochberg adjustment across three comparisons). **b** Venn diagrams of the significantly tightened modules by each intervention. For each set of the tightened modules, contingency table indicates the number of modules for which the other intervention groups exhibited significantly higher or lower mean of rank matching scores (RMSs) under the rank consensus than control group (i.e., similarly or dissimilarly changed module to the consensus group, respectively; two-sided Welch's *t*-tests after the Benjamini–Hochberg adjustment across six (two comparisons × three rank consensus) comparisons). **c**, **d** Sample RMS distributions for an example of the tightened modules (GO:0006511, ubiquitin-dependent protein catabolic process). Dashed line in **d** indicates the mean of RMSs for the rank consensus group (i.e., RCI). Data: the mean (dot) with 95% confidence interval (CI) (bar); $n = 12$ mice. *$P < 0.05$, **$P < 0.01$, ***$P < 0.001$ by two-sided Welch's *t*-tests after the Benjamini–Hochberg adjustment across three (**c**) or six (two comparisons × three rank consensus; **d**) comparisons.
