# Supplementary material for: Lifespan-extending interventions induce consistent patterns of fatty acid oxidation in mouse livers
Source: Commun Biol. 2023 Jul 22;6:768. doi: 10.1038/s42003-023-05128-y (PMC10363145; doi:10.1038/s42003-023-05128-y)
Supplement: Supplementary file 3 — Description of Additional Supplementary Files [file 42003_2023_5128_MOESM3_ESM.pdf]

Description of Additional Supplementary Files

File name: Supplementary Data 1.
Description: DIRAC analysis of the LC-M001 proteomics data using GOBP-defined modules. This .xlsx file contains the DIRAC results of the LCM001 proteomics data using GOBP-defined modules (Fig. 2, Supplementary Fig. 2), including metadata for the examined modules, group statistics (e.g., sample size, mean, s.e.m.), and test summary (e.g., degrees of freedom, test statistic, P-value). Descriptions about each sheet and each column are included in the README sheet.

File name: Supplementary Data 2.
Description: WGCNA of the LC-M001 proteomics data. This .xlsx file contains the WGCNA results of the LC-M001 proteomics data (Fig. 3a–e, Supplementary Fig. 3a–d), including metadata for the identified modules, group statistics (e.g., sample size, mean, s.e.m.), and test summary (e.g., degrees of freedom, test statistic, P-value). Descriptions about each sheet and each column are included in the README sheet.

File name: Supplementary Data 3.
Description: Enrichment analysis for the hub proteins of WGCNA-identified modules. This .xlsx file contains the overrepresentation test results for the enrichment of GOBP terms in the top 10% hub proteins of the WGCNA-identified modules (Fig. 3d, Supplementary Fig. 3c), including test summary (e.g., degrees of freedom, test statistic, P-value). Descriptions about each sheet and each column are included in the README sheet.

File name: Supplementary Data 4.
Description: DIRAC analysis of the LC-M001 proteomics data using WGCNA-identified modules. This .xlsx file contains the DIRAC results of the LCM001 proteomics data using WGCNA-identified Page 8 of 21 modules (Fig. 3f–i; Supplementary Fig. 3e, f), including metadata for the examined modules, group statistics (e.g., sample size, mean, s.e.m.), and test summary (e.g., degrees of freedom, test statistic, P-value). Descriptions about each sheet and each column are included in the README sheet.

File name: Supplementary Data 5.
Description: DIRAC analysis of the M001-related transcriptomics data. This .xlsx file contains the DIRAC results of the M001-related transcriptomics data using GOBP-defined modules (Fig. 4a, Supplementary Fig. 4), including metadata for the examined modules, group statistics (e.g., sample size, mean, s.e.m.), and test summary (e.g., degrees of freedom, test statistic, P-value). Descriptions about each sheet and each column are included in the README sheet.

File name: Supplementary Data 6.
Description: DIRAC comparison analysis between the LC-M001 proteomics and M001-related transcriptomics data. This .xlsx file contains the DIRAC comparison results between the LC-M001 proteomics and M001- related transcriptomics data using GOBP-defined modules (Fig. 4b–d, 5d, 5g), including metadata for the examined modules, group statistics (e.g., sample size, mean, s.e.m.), and test summary (e.g., degrees of freedom, test statistic, P-value). Descriptions about each sheet and each column are included in the README sheet.

File name: Supplementary Data 7.
Description: DIRAC analysis of the LC-M001 and LC-M004 proteomics data. This .xlsx file contains the DIRAC results of the LCM001 and LC-M004 proteomics data using GOBP-defined modules (Fig. 5a–c, e, f), including metadata for the examined modules, group statistics (e.g.,

sample size, mean, s.e.m.), and test summary (e.g., degrees of freedom, test statistic, P-value). Descriptions about each sheet and each column are included in the README sheet.

File name: Supplementary Data 8.
Description: Integrated analysis of the M001-related transcriptomics data with mouse GEM. This .xlsx file contains the flux comparison and overrepresentation test results for the shifted GEM subsystems using mouse GEM reconstruction with the M001-related transcriptomics data (Fig. 6), including metadata for the examined reactions, group statistics (e.g., sample size, mean, median, MAD), and test summary (e.g., degrees of freedom, test statistic, P-value). Descriptions about each sheet and each column are included in the README sheet.

File name: Supplementary Data 9.
Description: Source data. This .xlsx file contains all source data underlying Figures and Supplementary Figures of this paper (i.e., all exact values presented in the figures). Comments about each sheet are included in the README sheet.
