# Supplementary material for: Lifespan-extending interventions induce consistent patterns of fatty acid oxidation in mouse livers
Source: Commun Biol. 2023 Jul 22;6:768. doi: 10.1038/s42003-023-05128-y (PMC10363145; doi:10.1038/s42003-023-05128-y)
Supplement: Supplementary file 13 — Reporting Summary [file 42003_2023_5128_MOESM13_ESM.pdf]

nature portfolio

Corresponding author(s): Leroy Hood & Noa Rappaport

Last updated by author(s): Jun 30, 2023

# Reporting Summary

Nature Portfolio wishes to improve the reproducibility of the work that we publish. This form provides structure for consistency and transparency in reporting. For further information on Nature Portfolio policies, see our Editorial Policies and the Editorial Policy Checklist.

## Statistics

For all statistical analyses, confirm that the following items are present in the figure legend, table legend, main text, or Methods section.

| n/a | Confirmed | |
|---|---|---|
| ☐ | ☒ | The exact sample size (*n*) for each experimental group/condition, given as a discrete number and unit of measurement |
| ☐ | ☒ | A statement on whether measurements were taken from distinct samples or whether the same sample was measured repeatedly |
| ☐ | ☒ | The statistical test(s) used AND whether they are one- or two-sided<br>*Only common tests should be described solely by name; describe more complex techniques in the Methods section.* |
| ☐ | ☒ | A description of all covariates tested |
| ☐ | ☒ | A description of any assumptions or corrections, such as tests of normality and adjustment for multiple comparisons |
| ☐ | ☒ | A full description of the statistical parameters including central tendency (e.g. means) or other basic estimates (e.g. regression coefficient) AND variation (e.g. standard deviation) or associated estimates of uncertainty (e.g. confidence intervals) |
| ☐ | ☒ | For null hypothesis testing, the test statistic (e.g. *F*, *t*, *r*) with confidence intervals, effect sizes, degrees of freedom and *P* value noted<br>*Give P values as exact values whenever suitable.* |
| ☒ | ☐ | For Bayesian analysis, information on the choice of priors and Markov chain Monte Carlo settings |
| ☒ | ☐ | For hierarchical and complex designs, identification of the appropriate level for tests and full reporting of outcomes |
| ☐ | ☒ | Estimates of effect sizes (e.g. Cohen's *d*, Pearson's *r*), indicating how they were calculated |

*Our web collection on statistics for biologists contains articles on many of the points above.*

## Software and code

Policy information about availability of computer code

| | |
|---|---|
| Data collection | Mouse mass spectrometry (MS) data was collected using the Trans-Proteomic Pipeline (ref. 70). Peptide identification was performed by database searching with Comet (ref. 71). Peptide sequences were validated with PeptideProphet (ref. 72) and iProphet (ref. 73). Protein inference was performed with ProteinProphet (ref. 74). Protein quantification was performed using the top-3 method (ref. 75,76).<br>Mouse transcriptomics data was generated in the previous study (ref. 45), and its preprocessed data was kindly provided by Vadim N. Gladyshev (Harvard Medical School). |
| Data analysis | Differential Rank Conservation (DIRAC) analysis was performed using the reimplemented Python (version 3.7.6 or 3.9.7) code. Weighted Gene Coexpression Network Analysis (WGCNA) was performed using R WGCNA package (version 1.71). Flux Variability Analysis of genome-scale metabolic models (GEMs) was performed using the COBRA toolbox (version 3.0). The COBRA toolbox was implemented in MATLAB (R2019a), and academic licenses of Gurobi optimizer (version 7.5) and IBM CPLEX (version 12.7.1) were used to solve LP (Linear Programming) and MILP (Mixed Integer Linear Programming) problems.<br>All processing and null hypothesis testing were performed using Python (version 3.9.7) with Python NumPy (version 1.21.3), pandas (version 1.3.4), SciPy (version 1.7.1) and statsmodels (version 0.13.0) libraries, except for overrepresentation analysis using R (version 4.1.1) with R tidyverse (version 1.3.1) and clusterProfiler (version 4.2.2) packages.<br>Most results were visualized using Python (version 3.9.7) with Python matplotlib (version 3.4.3), seaborn (version 0.11.2), venn (version 0.1.3) libraries, while the results of enrichment analyses were visualized using R (version 4.1.1) with R ggplot2 (version 3.3.6) and enrichplot (version 1.14.2) packages.<br>Code used in this study is freely available on GitHub (https://github.com/longevity-consortium/SysBioM001Paper). |

For manuscripts utilizing custom algorithms or software that are central to the research but not yet described in published literature, software must be made available to editors and reviewers. We strongly encourage code deposition in a community repository (e.g. GitHub). See the Nature Portfolio guidelines for submitting code & software for further information.

## Data

Policy information about availability of data

All manuscripts must include a data availability statement. This statement should provide the following information, where applicable:

- Accession codes, unique identifiers, or web links for publicly available datasets
- A description of any restrictions on data availability
- For clinical datasets or third party data, please ensure that the statement adheres to our policy

The MS data of the LC-M001 and LC-M004 proteomics have been deposited to the ProteomeXchange Consortium via the PRIDE partner repository (PXD035255). Note that this data will be available after journal publication; for reviewing purpose, we provided a reviewer account information within the previous cover letter. The processed data of the M001-related transcriptomics was kindly provided by Vadim N. Gladyshev (Harvard Medical School), and raw data is available on the NCBI's Gene Expression Omnibus (GEO) repository (GSE131901). Source Data are provided with this paper (Supplementary Data 9).

## Research involving human participants, their data, or biological material

Policy information about studies with human participants or human data. See also policy information about sex, gender (identity/presentation), and sexual orientation and race, ethnicity and racism.

| | |
|---|---|
| Reporting on sex and gender | This study did not involve human participants, their data, or biological material. |
| Reporting on race, ethnicity, or other socially relevant groupings | This study did not involve human participants, their data, or biological material. |
| Population characteristics | This study did not involve human participants, their data, or biological material. |
| Recruitment | This study did not involve human participants, their data, or biological material. |
| Ethics oversight | This study did not involve human participants, their data, or biological material. |

Note that full information on the approval of the study protocol must also be provided in the manuscript.

# Field-specific reporting

Please select the one below that is the best fit for your research. If you are not sure, read the appropriate sections before making your selection.

☒ Life sciences ☐ Behavioural & social sciences ☐ Ecological, evolutionary & environmental sciences

For a reference copy of the document with all sections, see nature.com/documents/nr-reporting-summary-flat.pdf

# Life sciences study design

All studies must disclose on these points even when the disclosure is negative.

| | |
|---|---|
| Sample size | Sample size was not predetermined by statistical methods. In mouse experiments, mice were prepared based on practical feasibility. Because our primary questions in this study were about intervention effects, not sex-specific effects, we did not try to expand sample size per sex. This limitation was clearly described in the Result, Discussion, and Methods sections. |
| Data exclusions | In WGCNA, the analytes that were missing in 50% or more samples were eliminated, according to the default setting. In DIRAC analyses of mouse datasets, all the analytes that were not detected in one or more samples were eliminated during processing, because missingness needs to be resolved prior to DIRAC analysis. This is more conservative approach compared to imputation.<br>In the analyses using the M001-related transcriptomics data (ref. 45), only the 48 samples (acarbose, rapamycin, calorie restriction, and their corresponding control) were selected, and the remaining 30 samples were eliminated. It's because these eliminated intervention groups had the insufficient sample size for statistical power and robustness of the analyses.<br>All these exclusions were also described in the Result and/or Method sections. |
| Replication | In mouse experiments, all data were derived from independent mice; i.e., biological replicates. |
| Randomization | In mouse experiments, mice were randomly allocated to each intervention group while balancing sex and sample size. |
| Blinding | Because completely different researchers performed experiments, data generation, and data analysis independently, further blinding was not performed in this study. |

# Reporting for specific materials, systems and methods

We require information from authors about some types of materials, experimental systems and methods used in many studies. Here, indicate whether each material, system or method listed is relevant to your study. If you are not sure if a list item applies to your research, read the appropriate section before selecting a response.

## Materials & experimental systems

| n/a | Involved in the study |
| --- | --- |
| ☒ | ☐ Antibodies |
| ☒ | ☐ Eukaryotic cell lines |
| ☒ | ☐ Palaeontology and archaeology |
| ☐ | ☒ Animals and other organisms |
| ☒ | ☐ Clinical data |
| ☒ | ☐ Dual use research of concern |
| ☒ | ☐ Plants |

## Methods

| n/a | Involved in the study |
| --- | --- |
| ☒ | ☐ ChIP-seq |
| ☒ | ☐ Flow cytometry |
| ☒ | ☐ MRI-based neuroimaging |

# Animals and other research organisms

Policy information about studies involving animals; ARRIVE guidelines recommended for reporting animal research, and Sex and Gender in Research

**Laboratory animals**

Female and male genetically heterogenous UM-HET3 mice, which has unique genetic background but shared the same set of inbred grandparents (C57BL/6J, BALB/cByJ, C3H/HeJ, and DBA/2J), were used at 12 months in the LC-M001 experiment and at 6–8 months in the LC-M004 experiment.
The M001-related experiment was performed in the previous study (ref. 45). Each of the four groups (control, acarbose, rapamycin, and calorie restriction) consisted of three 6 months-old female, three 12 months-old female, three 6 months-old male, and three 12 months-old male UM-HET3 mice (n = 12 mice per group).

**Wild animals**

This study did not involve wild animals.

**Reporting on sex**

This study reports only the robust findings that are applicable to both sex. In all experiments, mice were prepared in a sex-balanced manner: n = 12 (6 female and 6 male) mice per group for the LC-M001 experiment and the M001-related experiment (Ref. 45) or n = 8 (4 female and 4 male) mice per group for the LC-M004 experiment. Acknowledging the limited sample size for statistical power and robustness of downstream analyses, we regressed out the potential effects of sex as the first preprocessing step for all analyses and pooled female and male samples per intervention throughout the current study. To verify the sex-pooled result, sex-stratified analysis was provided in Supplementary Fig. 1a. This point was explicitly described in the Result, Discussion, and Methods sections.

**Field-collected samples**

This study did not involve samples collected from the field.

**Ethics oversight**

All the animal protocols in the LC-M001 and LC-M004 experiments were approved by the University of Michigan's Institutional Animal Care and Use Committee (IACUC). In the M001-related experiment, the animal protocols as to the assessed four groups were approved by the IACUC at the University of Michigan.

Note that full information on the approval of the study protocol must also be provided in the manuscript.
